# Editorial: Neural mechanisms and clinical advances in binocular vision

**DOI:** 10.3389/fnins.2026.2003551

**Published:** 2026-10-14

**Authors:** Jameel Rizwana Hussaindeen, Ana Sanchez-Cano, Elvira Orduna-Hospital

**Affiliations:** 1 Department of Optometry and Vision Sciences, College of Health Sciences, Gulf Medical University, Ajman, United Arab Emirates; 2 Department of Applied Physics, University of Zaragoza, Zaragoza, Spain

**Keywords:** accommodation, binocular rivalry, binocular vision, normative data, stereopsis, strabismus, vergence, visual attention

Binocular vision is the ability to use both eyes together to perceive a single, clear and three-dimensional image of the visual environment (von Noorden and Campos, 2002). This requires the two eyes to be aligned on the object of regard through coordinated eye movements, with accommodation and vergence working together resulting in a clear and single image at all viewing distances (Scheiman and Wick, 2020). Fusion, however, depends on several variables, including but not limited to ocular alignment, the interaction between accommodation and vergence, and the capacity of the visual system to adapt to changes in any of these (von Noorden and Campos, 2002). Disruption at any level, whether motor, sensory or adaptive, results in the conditions seen routinely in clinical practice, such as strabismus, suppression, amblyopia, loss of stereopsis and non-strabismic binocular vision anomalies.

This Research Topic was conceived to bring together evidence from the laboratory and the clinic toward understanding the neural mechanisms of binocular vision and advancing its clinical management. Five articles were published in the Topic and are summarized in this editorial. Figure 1 gives a pictorial representation of how the articles relate to one another, from normative measurement, through neural mechanisms, to clinical classification and treatment.

**Figure 1:**
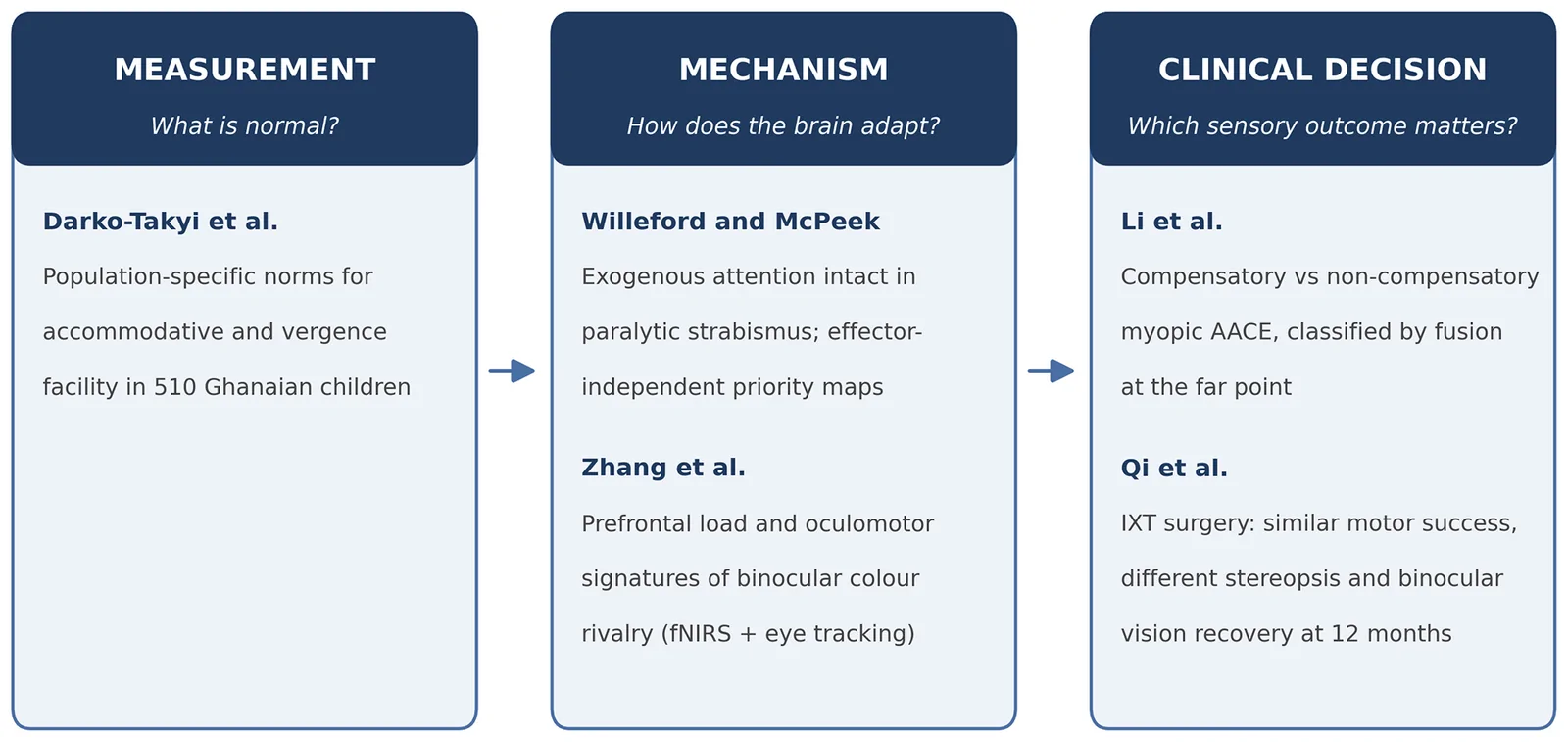
From measurement to mechanism to clinical decision-making: the proposed linkage between the five articles published in this Research Topic. The schematic was generated with the assistance of Claude (Anthropic).

Darko-Takyi et al. report normative data for accommodative facility and vergence facility in Ghanaian school children aged 8 to 17 years. From 2,300 children screened, 510 were asymptomatic and free of binocular vision anomalies and formed the normative sample. The median values were 13 cpm for monocular and binocular accommodative facility and 14 cpm for vergence facility. According to the authors, this is the first report of vergence facility norms from an African population. The spread of normal values was wide, and the authors recommend that the presence or absence of symptoms be considered when interpreting a measurement against the range. The values also differ from those reported in other ethnic groups, which emphasizes the need for population-specific normative data rather than reliance on norms developed in other populations.

Willeford and McPeek examined exogenous spatial attention in five patients with paralytic strabismus, comprising Duane retraction syndrome type I and acquired sixth nerve palsy. Earlier studies had reported no cueing effects in such patients and interpreted this as evidence that attention cannot be directed to a region of space the eyes cannot move to. Using a paradigm with multiple cue locations, a no-cue control condition, and both saccadic and keypress responses, the authors found that all five patients showed the expected pattern: faster responses to targets near the cue and slower responses to targets away from it. This was seen in both hemifields and with either eye viewing. The authors conclude that attention is altered rather than absent in paralytic strabismus and that the priority maps in premotor structures appear to operate independently of the effector. From a clinical standpoint, the findings caution against inferring a deficit in attention from a restriction in eye movement.

Zhang et al. studied binocular color rivalry in 16 healthy adults using simultaneous eye tracking and functional near-infrared spectroscopy. Color rivalry was associated with pupil dilation and increased prefrontal activation, a change in stimulus location was processed with less prefrontal involvement, and the combination of the two produced the strongest activation in BA10 and the widest variability in reaction time. Prefrontal oxyhaemoglobin was positively correlated with reaction time. These findings indicate that the prefrontal cortex is involved in resolving interocular conflict, and the oculomotor and haemodynamic measures described could be explored as markers in patients with suppression or unstable fusion.

Li et al. evaluated 105 myopic patients with acute acquired comitant esotropia and 107 myopic controls. Prolonged near work without spectacle correction was reported by 98% of the patients compared with 13% of the controls, whereas routine spectacle wear did not differ between the groups. The authors classified the patients according to whether fusion could be maintained at the far point without correction. The compensatory group (88%) had larger deviations at distance than at near, a larger deviation on the Maddox rod than on the cover test, and near-orthophoria at the far point, consistent with an adaptation to excessive convergence demand. The non-compensatory group was younger, less myopic, had larger angles of deviation and poorer stereopsis. Two points from this work are of practical value: the cover test may underestimate the deviation in the compensatory group, and near work without correction is a modifiable risk factor.

Qi et al. compared bilateral lateral rectus recession with unilateral recession-resection in 306 children with intermittent exotropia followed for 12 months. Motor success was similar between the two procedures (87.5% and 91.2%). The recession-resection group had better foveal stereopsis and binocular vision recovery, while the bilateral recession group showed greater improvement in sensory alignment on the synoptophore and a higher rate of early overcorrection. The study was retrospective and the groups differed at baseline, so these findings need to be confirmed in a randomized trial. The results nevertheless show that motor alignment and sensory recovery are separate outcomes.

Several questions remain for future exploration. Ethnicity-specific normative data are required and should be related to symptoms and treatment outcomes. The attention and rivalry paradigms in this Topic were tested in small samples of patients with paralytic strabismus and in healthy adults, and further studies are required in comitant strabismus, amblyopia and non-strabismic binocular vision anomalies, including convergence insufficiency, to determine whether these neural measures have diagnostic or prognostic value. The far point classification of acute acquired comitant esotropia needs further validation with objective measurement of near work. Prospective randomized clinical trials are warranted to compare surgical techniques, with well-defined clinical outcome measures, long-term follow-up, and endpoints based on binocular vision parameters.

We thank the authors for their contributions and the reviewers for their careful evaluation of each manuscript.
